# Sonic Hedgehog Signaling Regulates Hematopoietic Stem/Progenitor Cell Activation during the Granulopoietic Response to Systemic Bacterial Infection

**DOI:** 10.3389/fimmu.2018.00349

**Published:** 2018-02-26

**Authors:** Xin Shi, Shengcai Wei, Kevin J. Simms, Devan N. Cumpston, Thomas J. Ewing, Ping Zhang

**Affiliations:** ^1^Department of Integrative Medical Sciences, College of Medicine, Northeast Ohio Medical University, Rootstown, OH, United States; ^2^Department of Dermatology, Zhujiang Hospital, Southern Medical University, Guangzhou, China

**Keywords:** hedgehog signaling, hematopoietic stem cells, progenitor cells, the granulopoietic response, bacterial infection

## Abstract

Activation and reprogramming of hematopoietic stem/progenitor cells play a critical role in the granulopoietic response to bacterial infection. Our current study determined the significance of Sonic hedgehog (SHH) signaling in the regulation of hematopoietic precursor cell activity during the host defense response to systemic bacterial infection. Bacteremia was induced in male Balb/c mice *via* intravenous injection (i.v.) of *Escherichia coli* (5 × 10^7^ CFUs/mouse). Control mice received i.v. saline. SHH protein level in bone marrow cell (BMC) lysates was markedly increased at both 24 and 48 h of bacteremia. By contrast, the amount of soluble SHH ligand in marrow elutes was significantly reduced. These contrasting alterations suggested that SHH ligand release from BMCs was reduced and/or binding of soluble SHH ligand to BMCs was enhanced. At both 12 and 24 h of bacteremia, SHH mRNA expression by BMCs was significantly upregulated. This upregulation of SHH mRNA expression was followed by a marked increase in SHH protein expression in BMCs. Activation of the ERK1/2–SP1 pathway was involved in mediating the upregulation of SHH gene expression. The major cell type showing the enhancement of SHH expression in the bone marrow was lineage positive cells. Gli1 positioned downstream of the SHH receptor activation serves as a key component of the hedgehog (HH) pathway. Primitive hematopoietic precursor cells exhibited the highest level of baseline Gli1 expression, suggesting that they were active cells responding to SHH ligand stimulation. Along with the increased expression of SHH in the bone marrow, expression of Gli1 by marrow cells was significantly upregulated at both mRNA and protein levels following bacteremia. This enhancement of Gli1 expression was correlated with activation of hematopoietic stem/progenitor cell proliferation. Mice with Gli1 gene deletion showed attenuation in activation of marrow hematopoietic stem/progenitor cell proliferation and inhibition of increase in blood granulocytes following bacteremia. Our results indicate that SHH signaling is critically important in the regulation of hematopoietic stem/progenitor cell activation and reprogramming during the granulopoietic response to serious bacterial infection.

## Introduction

Primitive hematopoietic precursor cells, specifically hematopoietic stem cells (HSCs), are rare event cells in the bone marrow. In normal circumstances, most of these upstream precursors are maintained in the quiescent state with only a small proportion of them entering into cell cycling for self-renewal and/or proliferation (1). The homeostasis of HSC quiescence, self-renewal, proliferation, and differentiation secures maintaining the appropriate pool of HSCs while giving rise to all types of blood cells in the body. Our recent studies have revealed that primitive hematopoietic precursor cells in the adult bone marrow constitute a key component of the host immune defense system (2–4). During bacterial infection, marrow primitive hematopoietic precursor cells activate rapidly. While increasing proliferation to expand their own population in the bone marrow, these cells reprogram their transcriptional polarization toward enhancing commitment to granulocyte lineage (lin) development. Evoking the granulopoietic response to bacterial infection is critically important for boosting granulocyte production in order for reinforcing the phagocytic defense against invading pathogens. Currently, knowledge about cell signaling mechanisms underlying the activation and reprograming of primitive hematopoietic precursor cells in the process of the granulopoietic response to bacterial infection remains scant.

Hedgehog (HH) signaling has been reported to regulate stem cell activity during embryogenesis (5, 6) and in adulthood (7, 8). In mammals, three HH genes, including Sonic (*shh*), Indian (*ihh*), and Desert (*dhh*) HH, have been identified (9, 10). These gene products are initially 45-kDa precursor proteins, which are cleaved and then subjected to cholesterol and palmitoyl modification to produce a 19 kDa active N-terminal fragment (11–13). Among three HH proteins, Sonic hedgehog (SHH) is the best studied ligand (14). SHH molecules are expressed on the cell surface as transmembrane proteins (8, 15–17). SHH signals can be mediated through cell-to-cell contact between adjacent cells expressing the SHH receptor patched (PTCH) 1 and 2. Alternatively, NH2-terminal cleavage of SHH can generate a soluble SHH ligand to interact with distal cells expressing PTCH receptors. Binding of SHH ligand to PTCH abolishes PTCH-exerted repression of Smoothened (SMO) allowing SMO to become active, which activates SHH target gene transcription through the glioma-associated oncogene (Gli) transcription factor family (15–17). In the Gli family, Gli2 exists in both a full-length active form and a truncated repressor form (18, 19). Activated SMO prevents cell process of full-length Gli2 transcription factor into a truncated repressor, enabling Gli2 nuclear translocation to activate the transcription of target genes, particular Gli1 (19–22). Gli1 is a key transcriptional activator for expression of genes for cell proliferation and survival. Gli1 also activates Gli1 and PTCH (1 and 2) gene expression, which serves as positive and negative feedbacks of SHH signaling, respectively (17–23). SHH has been reported to promote primitive hematopoietic precursor proliferation and myeloid differentiation in mouse models (8, 24, 25). At the present time, nevertheless, it remains elusive if SHH–Gli1 signaling participates in the regulation of primitive hematopoietic precursor cell activation and reprograming in host defense against serious bacterial infection.

In this study, we employed both *in vivo* and *in vitro* model systems with manipulations of specific genes to determine the alteration of SHH–Gli1 signal system in bone marrow hematopoietic niche environment and in primitive hematopoietic cells. Our focus was on delineating the role of SHH–Gli1 signaling in the regulation of hematopoietic precursor cell activity during the granulopoietic response to systemic bacterial infection.

## Materials and Methods

### Animals

Male BALB/c mice (6–8 weeks old) were purchased from Charles River Laboratories (Wilmington, MA, USA). Male *Gli1*^null^ mice (6–8 weeks old, derived from STOCK Gli1tm2Alj/J Gli1lacZ mutant mice with 129S1/SvImJ strain background) were bred at Northeast Ohio Medical University Comparative Medicine Unit. Breeding pairs of *Gli1*^null^ (STOCK Gli1tm2Alj/J Gli1lacZ, Stock No. 008211) and the background control (129S1/SvImJ, Stock No. 002448) mouse strains were purchased from The Jackson Laboratory (Bar Harbor, ME, USA). All animals were housed in specific pathogen-free facilities with a 12 h light/dark cycle. This study was carried out in accordance with the recommendations of National Institutes of Health guidelines. The protocol was approved by the Institutional Animal Care and Use Committees of Northeast Ohio Medical University and Michigan State University prior to initiation of all experiments.

Bacteremia was induced in mice as described previously with minor modifications (4). Briefly, an intravenous (i.v. through the penile vein) injection of live *Escherichia coli* (*E. coli*, strain E11775 from the American Type Culture Collection, Rockville, MD; 5 × 10^7^ CFUs in 100 µl of pyrogen-free saline/mouse) was given to mice under anesthesia with inhalation of isoflurane (Henry Schein Animal Health, Dublin, OH, USA). Control mice received i.v. injection of an equal volume of pyrogen-free saline. In a subset of experiments, *E. coli* (5 × 10^7^ CFU in 50 µl pyrogen-free saline/mouse) or saline was i.v. injected into mice. Bromodeoxyuridine (5-bromo-2′-deoxyuridine or BrdU, BD Biosciences, San Diego, CA, USA; 1 mg in 100 µl of saline/mouse) was i.v. administered at the same time. Animals were sacrificed at scheduled time points as indicated in each figure legend in the Section “Results”.

At the time of sacrifice, a heparinized blood sample was obtained by cardiac puncture. White blood cells (WBCs) were quantified under a light microscope with a hemocytometer. Both femurs and tibias were collected. Bone marrow cells (BMCs) were flushed out from these bones with a total volume of 2 ml RPMI-1640 medium (Life Technologies, Grand Island, NY, USA) containing 2% bovine serum albumin (BSA, HyClone Laboratories, Logan, UT) through a 23-gage needle. BMCs were filtered through a 70-μm nylon mesh (Sefar America Inc., Kansas City, MO, USA). Contaminating erythrocytes in BMC samples were lysed with RBC lysis solution (Qiagen Sciences, Germantown, MD). Nucleated BMCs were washed with RPMI-1640 medium containing 2% BSA and then quantified under a light microscope with a hemocytometer. For determination of SHH level in bone marrow elute and nucleated BMC lysate samples, collected femurs, and tibias from each mouse were flushed with a total volume of 0.5 ml of phosphate-buffered saline (PBS, Life Technologies Co, Grand Island, NY, USA) through a 23-gage needle. Bone marrow elute samples were filtered through a 70-μm nylon mesh. After centrifugation at 500 × *g* for 5 min, bone marrow eluate (supernatant) samples were collected. Contaminating erythrocytes in the remaining BMC samples were lysed with RBC lysis solution as above. After washing twice with PBS, nucleated BMCs were collected. BMC lysate samples were prepared by lysing cells with a lysing buffer (10 mM Tris–HCl buffer containing 1% Triton X-100, 5 mM EDTA, 50 mM NaCl, 30 mM sodium pyrophosphate, 2 mM sodium orthovanadate, 1 mM PMSF, 50 mM sodium fluoride, 5 mg/ml aprotinin, 5 mg/ml pepstatin, and 5 mg/ml leupeptin, pH 7.6). After centrifugation at 10,000 × *g* for 10 min at 4°C, the supernatant of BMC lysate sample was collected. Bone marrow eluate and cell lysate samples were stored at −80°C till determination of SHH level.

### Preparation of Bacteria

For each experiment, a frozen stock culture of *E. coli* was added to tryptic soy broth and incubated for 18 h at 37°C in an orbital shaker. Bacteria were collected and washed twice with PBS. Suspension of bacteria in saline at appropriate concentrations was prepared based on its optical density at 600 nm. Actual numbers of viable bacteria were verified by standard plate counts of the bacterial suspensions on MacConkey agar plates following overnight incubation at 37°C.

### Culture of Primary Mouse BMCs

Isolated mouse BMCs were suspended in StemSpan serum-free medium (StemCell Technologies, Vancouver, BC, Canada) containing 20% mouse plasma and then plated into 24-well tissue culture plates with 500 µl of cell suspension (containing 5 × 10^6^ cells) per well. Culture of cells was conducted without or with lipopolysaccharide (LPS, *E. coli* 0111:B4, 20 ng/ml, Sigma-Aldrich Co., LLC, St. Louis, MO, USA) stimulation in the absence and presence of specific mitogen-activated protein kinase kinase1/2 (MEK1/2) inhibitor PD98059 (25 µM, LC Laboratories, Woburn, MA, USA) for 18 h.

### Determination of SHH Level with ELISA

Sonic hedgehog level in bone marrow elute and cell lysate samples was determined with enzyme-linked immunosorbent assay (ELISA) using the Mouse Shh-N ELISA Kit (Abcam, Cambridge, MA, USA) and the protocol provided by the manufacturer.

### Determination of Protein Content with BCA Protein Assay

Protein content in biological samples was determined using the BCA Protein Assay Kit (Thermo Fisher Scientific, Rockford, IL, USA) with the protocol provided by the manufacturer.

### Flourochrome Conjugation of Antibody

Flourochrome labeling of anti-human/mouse Gli1 antibody (Clone #388516, R&D Systems, Minneapolis, MN, USA) and the matched isotype control antibody (Clone # 54447, R&D Systems) was performed using DyLight 405 Microscale Antibody Labeling Kit (Thermo Fisher Scientific) with the protocol provided by the manufacturer.

### Flow Cytometric Analysis and Cell Sorting

Cell phenotype, cell membrane expression of SHH, and intracellular expression of specificity protein 1 (SP1) as well as Gli1 was determined with flow cytometry as previously described (2–4). Briefly, nucleated BMCs or WBCs suspended in RPMI-1640 containing 2% BSA (1 × 10^6^ cells in 100 µl medium) were added with a mixed panel of biotinylated anti-mouse lin markers [10 µg/ml of each antibody against CD3e (clone 145-2C11), CD45R/B220 (clone RA3-6B2), CD11b (Mac-1, clone M1/70), TER-119 (clone TER-119)] with or without granulocyte differentiation antigen-1 (Gr-1 or Ly-6G/Ly-6C, clone RB6-8C5), or isotype control antibodies (clones A19-3, R35-95, A95-1) (BD Biosciences). Following incubation for 20 min at 4°C, flourochrome-conjugated streptavidin, anti-mouse stem cell growth factor receptor (c-kit or CD117, clone 2B8) and anti-mouse stem cell antigen-1 (anti-Sca-1, Ly-6A/E, clone D7) without or with anti-Gr1 (Ly-6G, clone 1A8), or the matched isotype control antibodies (BD Biosciences) were added into the incubation system at the final concentration of 10 µg/ml for each agent. Samples were further incubated in the dark for 20 min at 4°C. Antibody-stained cells were then washed with cold PBS containing 2% BSA. For measuring cell BrdU incorporation, cells were further processed using a BD BrdU Flow Kit (BD Biosciences) with the procedure provided by the manufacturer. For measuring cell expression of SHH and SP1, cells were further processed to make both cell membrane and nuclear membrane permeable for antibody using the procedure (without the step of DNA digestion with DNase) provided by BD BrdU Flow Kit (BD Biosciences). Permeablized cells were incubated with 10 µg/ml of anti-human/mouse SHH (Clone E1, Santa Cruz Biotechnology, Inc., Dallas, TX, USA) and anti-human/mouse SP1 antibody (Clone E3, Santa Cruz Biotechnology, Inc.), respectively, in the dark for 20 min at room temperature. Each cell sample was then added with 10 µg/ml of the corresponding flourochrome-conjugated second antibody [polyclonal goat anti-mouse IgG (H + L), Life Technologies, Eugene, OR, USA and anti-mouse IgG2a, clone R-1915, BD Biosciences]. The cells were further incubated in the dark for 20 min at room temperature. The background staining control samples were incubated with the respective flourochrome-conjugated second antibody only. For determining cell expression of Gli1, permeablized cells were incubated with 10 µg/ml of flourochrome-conjugated anti-human/mouse Gli1 antibody (Clone #388516, R&D Systems) and the isotype control antibody (Clone # 54447, R&D Systems), respectively, in the dark for 20 min at room temperature. At the end of the staining procedure, cells were washed with the washing buffer provided with the BD BrdU Flow Kit (BD Biosciences) and then suspended in 0.5 ml of PBS containing 1% paraformaldehyde. Analysis of cell phenotypes, BrdU incorporation, expression of SHH, SP1, and Gli1 was performed on a FACSAria Fusion flow cytometer with FACSDiva software (Becton Dickinson, San Jose, CA, USA). Cell populations of interest were gated based on their marker or marker combinations. Depending on the cell types being analyzed, the number of cells acquired in each sample was in the range of 5,000–300,000. In a subset of experiments, gated bone marrow lin^−^c-kit^+^ cells were sorted with FACSAria Fusion flow cytometer (Becton Dickinson).

### Granulocyte-Macrophage Colony-Forming Unit (CFU-GM) Assays

Granulocyte-macrophage colony-forming unit assays of freshly sorted bone marrow lin^−^c-kit^+^ cells were performed by culturing the cells in Methocult GF M3534 medium (StemCell Technologies, Vancouver, BC, Canada) in the absence and presence of recombinant murine SHH (200 ng/ml, eBioscience/Thermo Fisher Scientific). One milliliter of Methocult GF M3534 medium containing 100 cells was plated to a 35 mm NunclonTM dish (Nunc, Rodkilde, Denmark). The cultures were conducted for seven days at 37°C in an atmosphere of 5% CO2. Colonies containing 50 or more cells were then enumerated.

### Western Blot Analysis

Western blot analysis of phosphorylated extracellular signal-regulated kinase 1/2 (phospho-ERK1/2) and total ERK1/2 protein expression by cells was performed using the protocol reported previously (4, 26, 27) with minor modifications. Protein was extracted from nucleated BMCs with the lysis buffer described above. Protein concentration was determined using BCA protein assay kit (Thermo Fisher Scientific). Thirty micrograms of protein sample were resolved using the 12% SDS-PAGE ready gel (Bio-Rad Laboratories, Hercules, CA, USA) and transferred to a polyvinylidene difluoride membrane (Bio-Rad Laboratories). The membrane was blocked with 5% milk in tris-buffered saline (Bio-Rad Laboratories) containing 0.1% Tween 20 (Sigma-Aldrich Co.) (TBST buffer) and hybridized sequentially with the primary antibody against phospho-ERK1/2 (anti-mouse phospho-p44/42 MAPK Thr202/Tyr204) and the corresponding HRP-conjugated secondary antibody (Cell Signaling Technology). Determination of the bound antibody was conducted using Amersham ECL Prime Western blotting Detection System (GE Healthcare, Piscataway, NJ) and imaged using Amersham Imager 600 (GE Healthcare Biosciences AB, Uppsala, Sweden). The membrane was stripped with Re-Blot Plus Mild Antibody Stripping Solution (EMD Millipore Cop., Billerica, MA, USA) and then re-probed sequentially with rabbit anti-mouse total ERK1/2 or anti-β-actin antibody (Cell Signaling Technology) and the corresponding HRP-conjugated goat anti-rabbit IgG to determine total ERK1/2 or anti-β-actin content, respectively. Semi-quantification was performed using the ImageJ software. Data are presented as the normalized intensity ratio of the detected protein band versus the loading reference (total ERK1/2 or β-actin) band in the same sample.

### Real-time RT-PCR Determination

Total RNA samples were prepared from nucleated BMCs and sorted marrow lin^−^c-kit^+^ cells with TRIzol reagent (Thermo Fisher Scientific) and RNeasy Plus Mini Kit (Qiagen, Valencia, CA, USA) using procedures provided by the manufacturers. Real-time RT-PCR analysis of mRNA expression by cells was performed as reported previously (28). Each RNA sample was subjected to 2-step real-time RT-PCR using iScriptTM Reverse Transcription Supermix kit and SsoFastTM EvaGreen^®^ Supermix kit (Bio-Rad Laboratories), respectively, on the CFX96TM Real-Time System (Bio-Rad Laboratories). The amplification primer pairs were as follows:

SHH
Forward 5′-TCCAAAGCTCACATCCACTG
Reverse 5′-CGTAAGTCCTTCACCAGCTTG
Gli1
Forward 5′-TTGTGGGAGGGAAGAAACCG
Reverse 5′-AGCCAGATCCATATGCTGCC
18SrRNA
Forward 5′-ATTCGAACGTCTGCCCTATAA
Reverse 5′-GTCACCCGTGGTCACCATG

These sets of primers were designed using Primer Express software (Life Technologies Co.). The expression of SHH and Gli1mRNA was determined by normalizing the cycle threshold number of their individual mRNA with that of 18S rRNA in each sample. Changes in specific gene mRNA expression by cells from groups with different treatments are expressed as fold alterations over the baseline expression by cells from the corresponding control group.

### Reporter Gene Analysis of Murine SHH Promoter Activity

HEK 293T cells (ATCC CRL-11268, American Type Culture Collection) were cultured in Opti-MEM reduced serum medium (Thermo Fisher Scientific) and transfected with pGL4.20[luc2/Puro] promoter reporter vector (Promega, Madison, WI, USA) containing murine shh promoter sequence [240 bp (from −258 to − 497) including 10 SP1 binding sites] in the absence and presence of co-transfection with SP1 expression vector (pUNO1-mSP1 vector, InvivoGen, San Diego, CA, USA). The activity of shh promoter in transfected HEK 293 T cells were determined following culture of cells for 48 h with the Dual-Luciferase^®^ Reporter (DLR™) Assay System on a Glomax 96 Microplate Luminometer (Promega).

### Statistical Analysis

Data are presented as mean ± SEM. The sample size is indicated in each figure legend. Statistical analysis was conducted using Student’s *t*-test for comparison between two groups and one-way ANOVA followed by Student–Newman–Keuls test for comparison among multiple groups. Difference with statistical significance is accepted at *p* < 0.05.

## Results

### Upregulation of SHH Expression by BMCs in Response to Bacteremia

**Figure 1:**
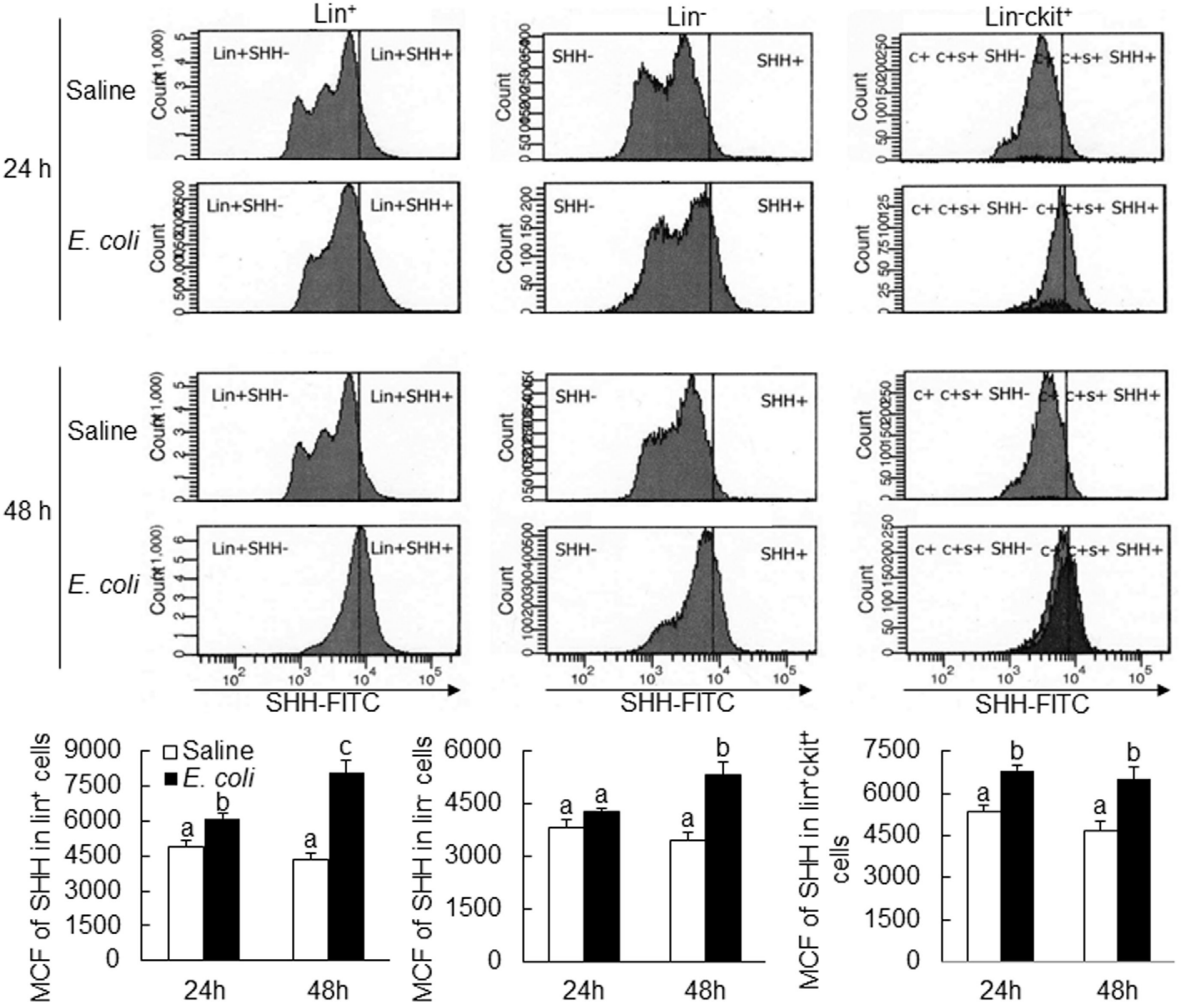
Changes in Sonic hedgehog (SHH) expression by bone marrow cells following systemic *E. coli* infection. Saline: i.v. injection of saline; *E. coli*: i.v. injection of *E. coli*; MCF, mean channel fluorescent intensity. Data are presented as mean ± SEM. *N* = 5. Bars with different letters in each panel are statically different (*p* < 0.05).

**Figure 2:**
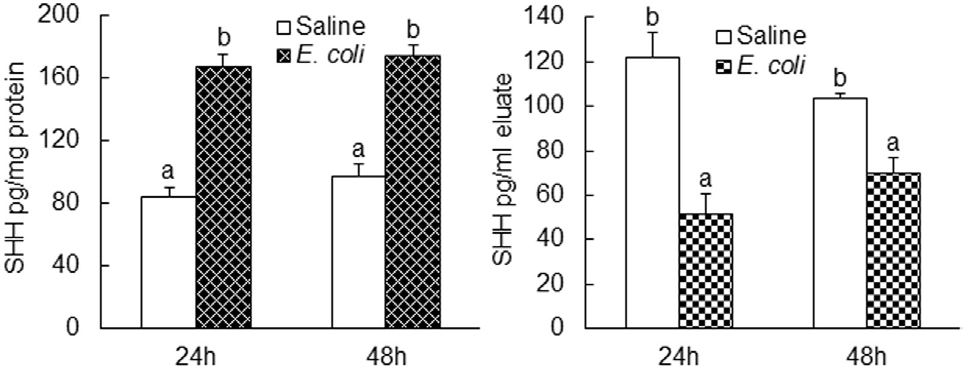
Changes in Sonic hedgehog (SHH) protein level in bone marrow cells lysate and bone marrow elute samples following systemic *E. coli* infection. Saline: i.v. injection of saline; *E. coli*: i.v. injection of *E. coli*. Data are presented as mean ± SEM. *N* = 5. Bars with different letters in each panel are statically different (*p* < 0.05).

**Figure 3:**
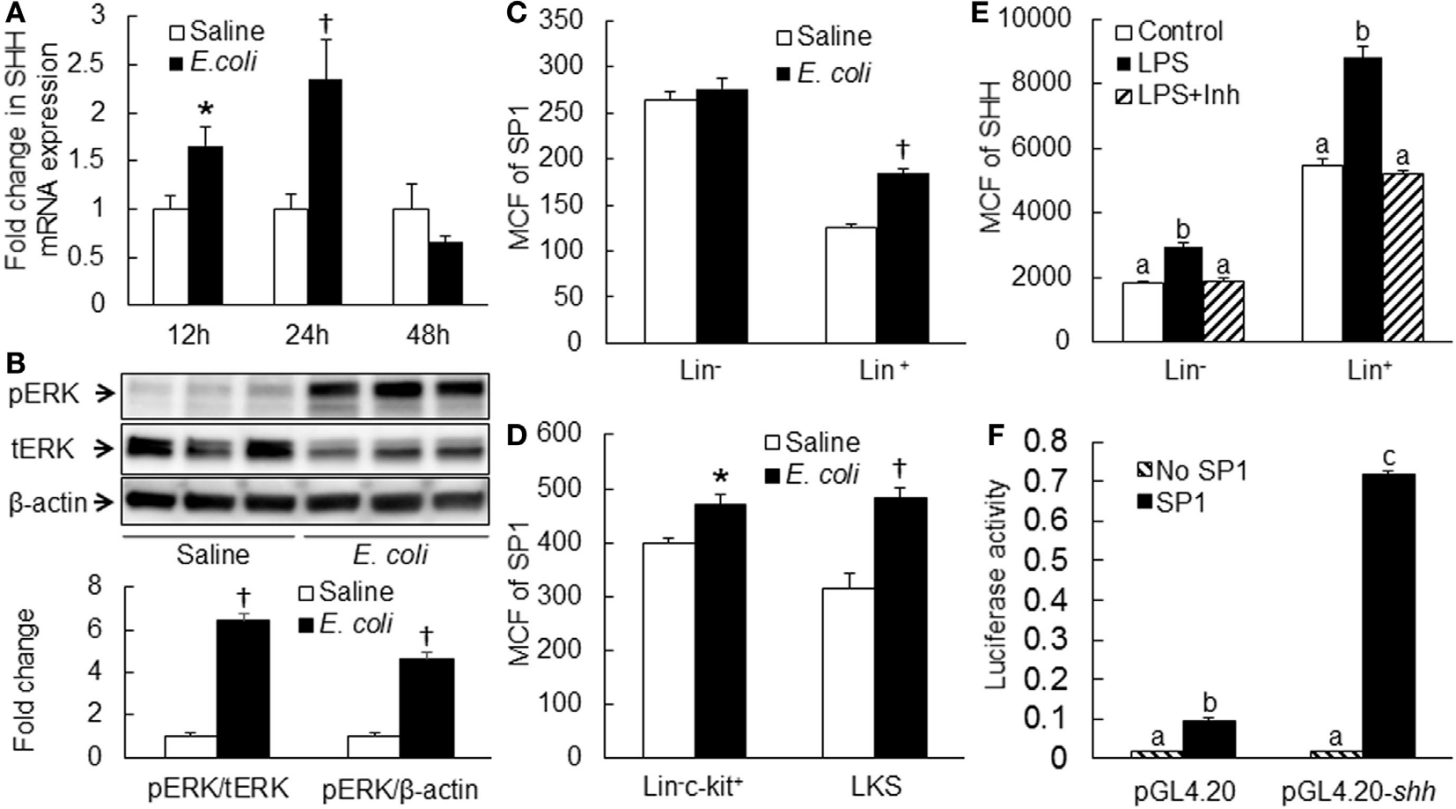
**(A)** Changes in Sonic hedgehog (SHH) mRNA expression by bone marrow cells (BMCs) following systemic *E. coli* infection. Saline: i.v. injection of saline; *E. coli*: i.v. injection of *E. coli*. Data are mean ± SEM. *N* = 4–9. *p < 0.05 compared to Saline group; †*p* < 0.01 compared to Saline group. **(B)** Activation of ERK1/2 in BMCs at 8 h of systemic *E. coli* infection. Saline: i.v. injection of saline; *E. coli*: i.v. injection of *E. coli*; pERK: phospho-ERK1/2; tERK: total ERK1/2. Data in the bar figure panel are mean ± SEM. *n* = 5. † *p* < 0.01 compared to Saline group. **(C,D)** Changes in SP1 expression by BMCs at 18 h of systemic *E. coli* infection. Saline: i.v. injection of saline; *E. coli*: i.v. injection of *E. coli*; MCF: mean channel fluorescent intensity. Data are mean ± SEM. N = 5. *p < 0.05 compared to Saline group; †*p* < 0.01 compared to Saline group. **(E)** Block of lipopolysaccharide (LPS)-induced SHH expression by BMCs by PD0325901. Control, control cell culture; LPS, LPS stimulation; LPS + Inh, LPS stimulation in the presence of PD0325901. Data are mean ± SEM. *N* = 5. Bars with different letters in each cell subpopulation are statistically different (*p* < 0.05). **(F)** SP1-induced enhancement of *shh* promoter activity in cultured HEK293 T cells. No SP1: cell transfected with control vector; SP1: cell transfected with SP1 expression vector; pGL4.20: cells transfected with control pGL4.20 promoter reporter vector; pGL4.20-shh: cells transfected with pGL4.20 promoter reporter vector containing murine shh promoter sequence. Data are mean ± SEM. *N* = 6. *p < 0.05 compared to the corresponding group transfected with control vector without SP1 gene.

Flow cytometric analysis showed that cells in marrow lin^+^ and lin^−^c-kit^+^ subpopulations significantly increased their expression of SHH 24 and 48 h following i.v. challenge with *E. coli* (Figure 1). Marrow lin^−^ cells also exhibited a significant increase in SHH expression 48 h following initiation of bacteremia. This increase in cell expression of SHH in response to systemic bacterial infection was validated by ELISA analysis (Figure 2). In association with the increase in SHH protein expression, SHH mRNA expression by BMCs was markedly upregulated 12 and 24 h following bacteremia (Figure 3A), indicating that the regulation at the transcriptional level was involved in the increase in SHH ligand expression by marrow cells in response to *E. coli* bacteremia. In contrast to the increase in cell-associated SHH level, the level of soluble SHH in bone marrow elute samples was markedly reduced at both 24 and 48 h post i.v. *E. coli* challenge (Figure 2), suggesting that either the release of SHH ligand by BMCs was reduced or cell binding of soluble SHH ligand in the hematopoietic niche environment was enhanced or possibly both following the systemic bacterial infection.

### Activation of ERK1/2-SP1 Signaling in Mediating Upregulation of SHH Expression

Analysis of murine *shh* promoter sequence using the AliBaba2.1 database predicted 14 Sp1 transcription factor binding sites with the identity of sequence greater than 80%. Toll-like receptor 4 (TLR4)-ERK1/2 signaling promotes SP1 expression (29, 30). We, therefore, determined if ERK-SP1 signaling was involved in the regulation of SHH expression during the host response to bacteremia. As shown in Figure 3B, phospho-ERK1/2 level in BMCs was markedly increased at the early stage (8 h) of bacteremia. Flow cytometric analysis showed that SP1 expression in marrow lin^+^, lin^−^c-kit^+^ and lin^−^c-kit^+^Sca-1^+^ (LKS, a cell population enriched with HSCs) cells was significantly upregulated 18 h post i.v. challenge with *E. coli* (Figures 3C,D). In the *in vitro* culture system, stimulation with LPS caused a significant increase in SHH expression by cultured BMCs (Figure 3E). This LPS-stimulated increase in SHH expression was abolished by specific MEK1/2 inhibitor PD98059. Reporter gene analysis demonstrated that SP1 markedly activated murine SHH promoter-driven luciferase expression or activity in HEK 293T cells (Figure 3F).

### Baseline Gli1 Expression by BMC Subpopulations

**Figure 4:**
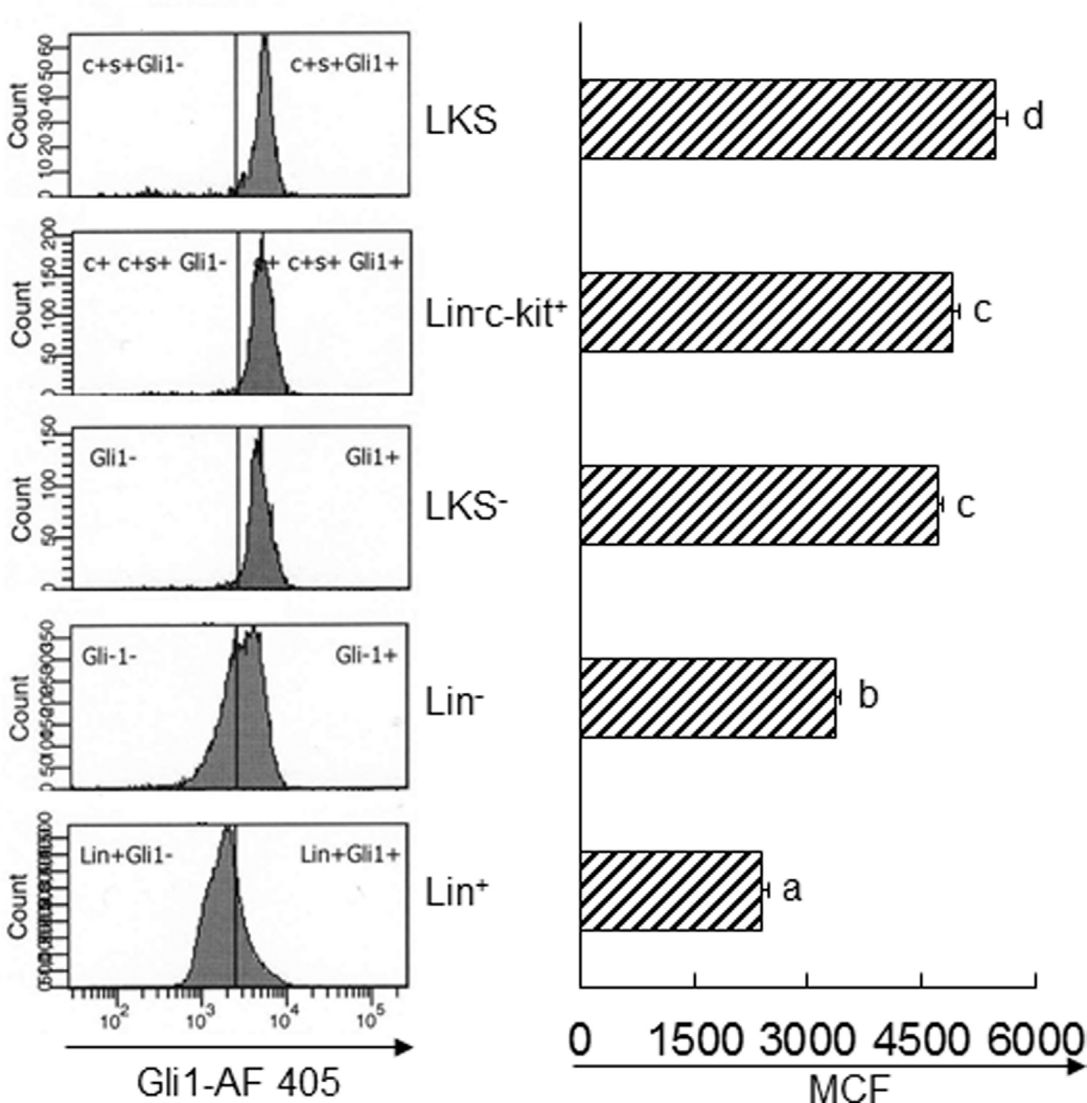
Baseline expression of Gli1 by subtypes of bone marrow cells. Data are presented as mean ± SEM. *N* = 4. Bars with different letters are statistically different (*p* < 0.05).

Activation of Gli1 expression by target cells responding to the SHH ligand stimulation is a key step in the SHH signal pathway, which promotes expression of downstream genes for modification of cell activities. We measured the level of Gli1 expression by mouse BMC subpopulations at different stages of differentiation and observed that marrow LKS cells expressed the highest level of Gli1 (Figure 4). Gli1 expression reduced with the maturation of hematopoietic cells. Lin^+^ cells expressed the lowest level of Gli1. These data suggest that primitive hematopoietic precursor cells, particularly HSCs, might be the most active cells responding to the stimulation of SHH ligand under the homeostatic circumstance.

### Increase in Gli1 Expression in BMCs following Bacteremia

**Figure 5:**
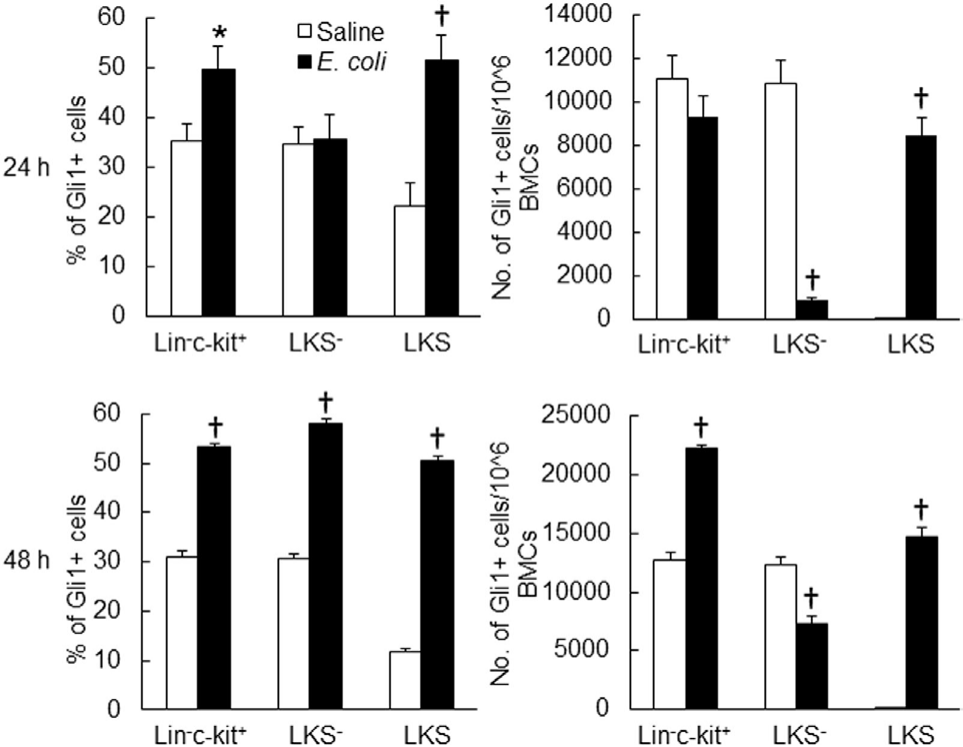
Changes in Gli1^+^ cells in marrow lin^−^ckit^+^, LKS^−^, and LKS cell subpopulations 24 and 48 h following systemic *E. coli* infection. Saline: i.v. injection of saline; *E. coli*: i.v. injection of *E. coli*. Data are mean ± SEM. *N* = 5–7. *p < 0.05; †*p* < 0.01 compared to the corresponding Saline group.

**Figure 6:**
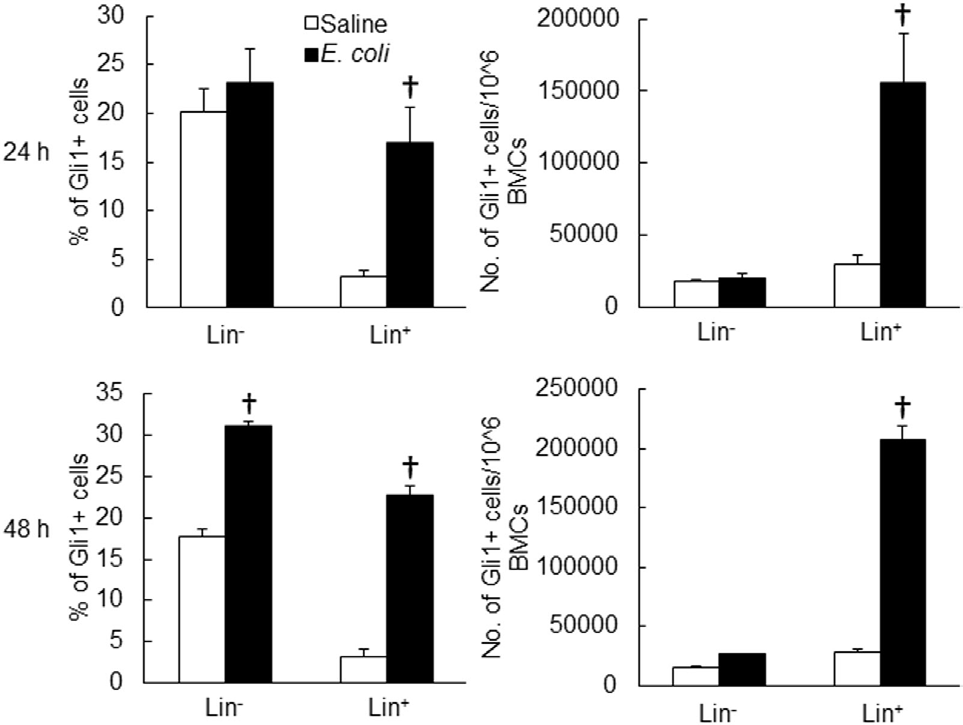
Changes in Gli1^+^ cells in marrow lin^−^ and lin^+^ cell subpopulations 24 and 48 h following systemic *E. coli* infection. Saline: i.v. injection of saline; *E. coli*: i.v. injection of *E. coli*. Data are mean ± SEM. *N* = 5–7. †*p* < 0.01 compared to the corresponding Saline group.

**Figure 7:**
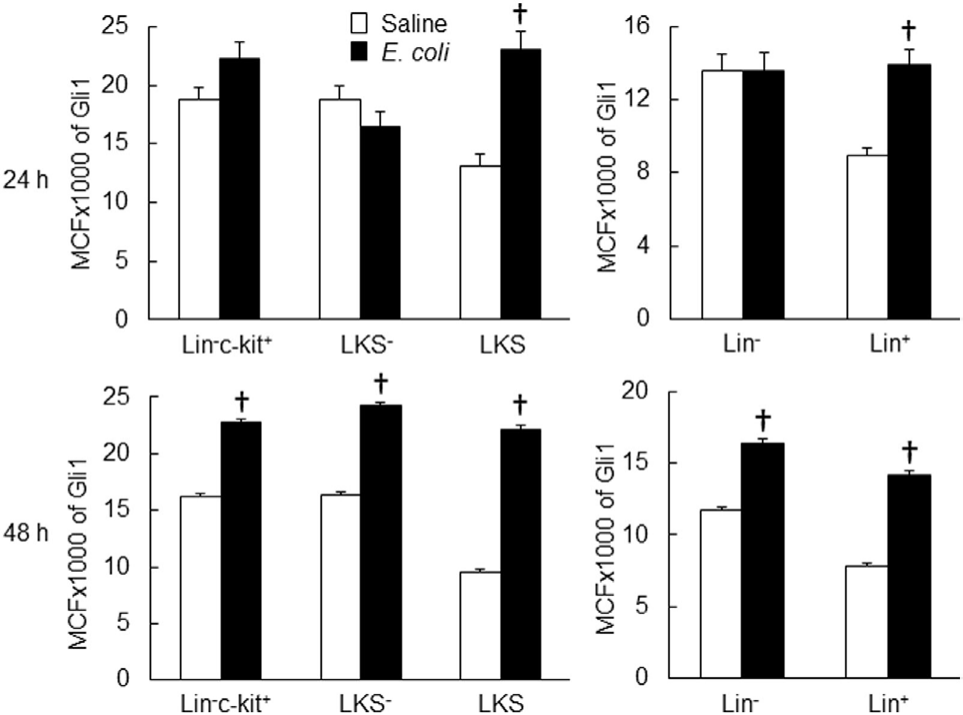
Changes in mean channel fluorescent intensity (MCF) of Gli1 expression by marrow cell subpopulations 24 and 48 h following systemic *E. coli* infection. Saline: i.v. injection of saline; *E. coli*: i.v. injection of *E. coli*. Data are mean ± SEM. *N* = 5–7. †*p* < 0.01 compared to the corresponding Saline group.

**Figure 8:**
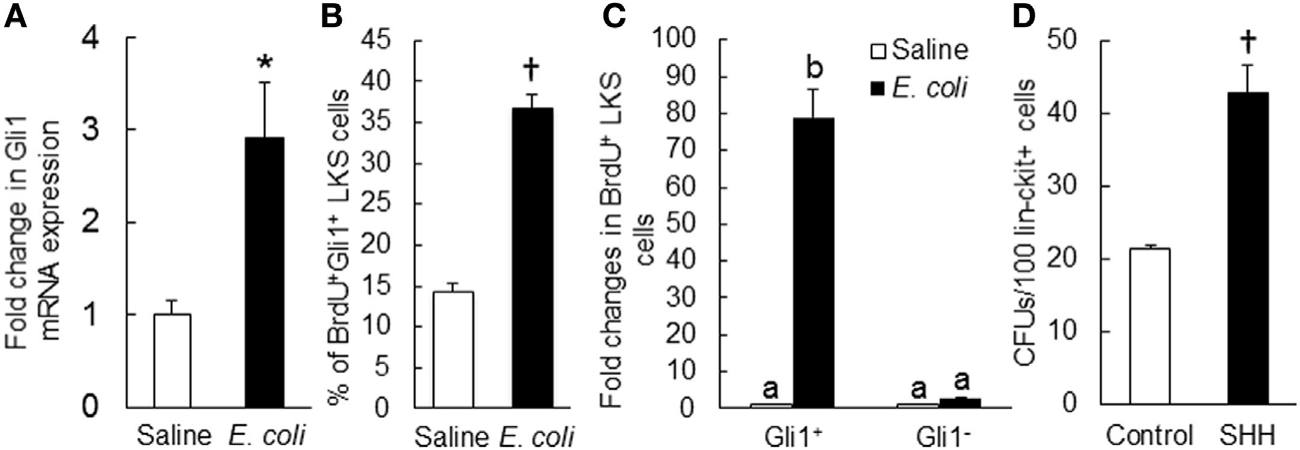
**(A)** Change in Gli1 mRNA expression by marrow lin^−^ckit^+^ cells at 24 h of systemic *E. coli* infection. Saline: i.v. injection of saline; *E. coli*: i.v. injection of *E. coli*. Values are mean ± SEM. *N* = 3. *p < 0.05 compared to the Saline group. **(B)** Change in percent of BrdU^+^Gli1^+^ LKS cells in the bone marrow and **(C)** Fold changes in the number of proliferating (BrdU^+^) LKS cells with and without Gli1 expression in the bone marrow at 24 h of systemic *E. coli* infection. Saline: i.v. injection of saline; *E. coli*: i.v. injection of *E. coli*. Data are mean ± SEM. *N* = 4 in each group. †*p* < 0.01 compared to the corresponding Saline group in **(B)**; bars with different letters in **(C)** are statistically different (*p* < 0.05). **(D)** Sonic hedgehog (SHH)-stimulated increase in granulocyte-macrophage colony-forming unit (CFU-GM) formation in cultured marrow lin^−^ckit^+^ cells. Control, without SHH stimulation; SHH, with SHH stimulation. Data are mean ± SEM. *N* = 4. †*p* < 0.01 compared to the control group.

Twenty-four hours following bacteremia, the percentage of Gli1^+^ cells was significantly increased in LKS, lin^−^c-kit^+^ and lin^+^ cell subpopulations (Figures 5 and 6). The numbers of Gli1^+^LKS and Gli1^+^lin^+^ cells in BMCs were significantly increased. Whereas the number of Gli1 positive lin^−^c-kit^+^Sca-1^−^ cells (LKS^−^ cells, an enriched myeloid progenitor cell population downstream of LKS cells) in BMCs was markedly reduced (Figure 5). Mean channel fluorescent intensity (MCF, reflecting the level of specific antigen in the gated cell population) of Gli1 was significantly elevated in LKS and lin^+^ cell subpopulations 24 h following bacteremia (Figure 7). Real-time RT-PCR determination showed that Gli1 mRNA expression was significantly upregulated in lin^−^c-kit^+^ cells 24 h following bacteremia (Figure 8A). By 48 h of bacteremia, the percentage of Gli1^+^ cells was significantly increased in LKS, LKS^−^, lin^−^c-kit^+^, lin^−^, and lin^+^ cell subpopulations (Figures 5 and 6). The numbers of Gli1^+^LKS, Gli1^+^lin^−^c-kit^+^, and Gli1^+^lin^+^ cells in BMCs were also significantly increased. However, the number of Gli1^+^LKS^−^ cells in BMCs remained significantly reduced (Figure 5). MCF of Gli1 was significantly elevated in LKS, LKS^−^, lin^−^c-kit^+^, lin^−^, and lin^+^ cell subpopulations 48 h following bacteremia (Figure 7).

### SHH–Gli1 Signaling in Activation of Primitive Hematopoietic Precursor Cells

To determine the role of SHH–Gli1 signaling in the activation and reprograming of primitive hematopoietic precursor cells during the granulopoietic response to systemic bacterial infection, we conducted *in vivo* cell BrdU incorporation and *in vitro* CFU-GM forming activity experiments. As shown in Figure 8B, the percentage of BrdU^+^Gli1^+^ cells in LKS cell subpopulation was significantly increased 24 h following bacteremia. The interesting observation was that the increase in BrdU incorporation into LKS cells following bacteremia was essentially in those cells expressing Gli1 (Figure 8C). Gli1^−^LKS did not show any increase in BrdU incorporation. These data suggest that upregulation of Gli1 expression may play a critical role in LKS activation of proliferation during the host response to bacteremia. *In vitro* culture of sorted marrow lin^−^c-kit^+^ cells in Methocult GF M3534 medium showed that stimulation with recombinant murine SHH ligand significantly increased CFU-GM forming activity of lin^−^c-kit^+^ cells (Figure 8D), indicating the enhancement of these hematopoietic precursor cell commitment toward myeloid differentiation and granulocyte lin development in response to SHH stimulation.

### Impairment of the Granulopoietic Response to Bacteremia with Gli1 Gene Deletion

**Figure 9:**
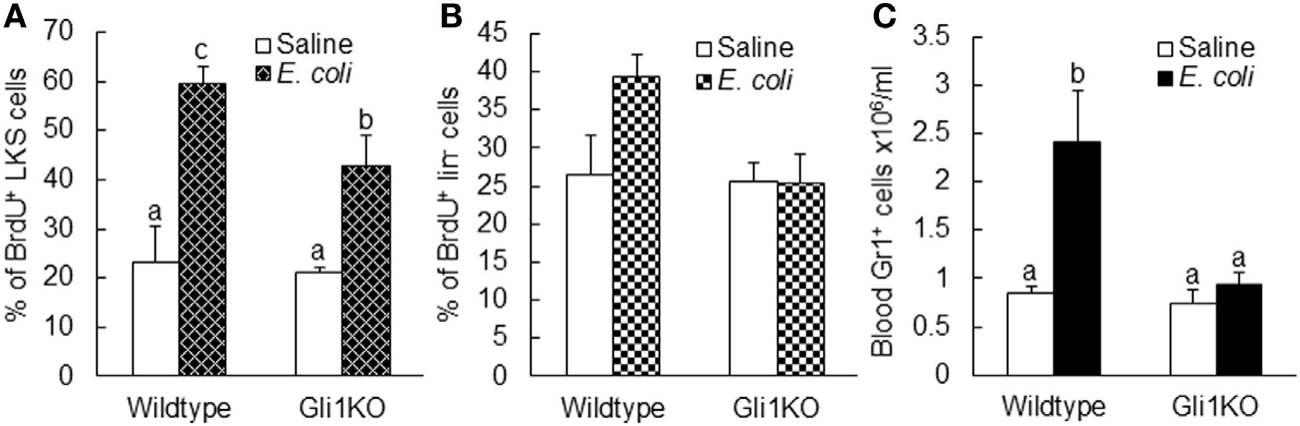
Alteration of BrdU incorporation into marrow LKS **(A)** and lin^−^ **(B)** cells as well as the number of circulating granulocytes **(C)** 24 h after i.v. challenge with *E. coli* in mice with *Gli1* gene deletion. Data are presented as mean ± SEM. *N* = 5–6. Bars with different letters in each panel are statistically different (*p* < 0.05).

To further verify the role of SHH–Gli1 signaling in the activation of primitive hematopoietic precursor cells during the granulopoietic response to serious bacterial infection, we employed an *in vivo* model of mice with *Gli1* deletion. As shown in Figure 9A, the activity of BrdU incorporation into LKS cells was significantly increased in wild-type mice 24 h following bacteremia. *Gli1* deletion did not affect the baseline activity of BrdU incorporation into LKS cells. However, bacteremia-induced activation of BrdU incorporation into LKS cells was significantly attenuated in mice with *Gli1* deletion. The activity of BrdU incorporation into marrow lin^−^ cells was also tended to increase (though did not reach the static significance) in response to bacteremia in wild-type mice (Figure 9B). This tendency was not seen in mice with *Gli1* deletion. Furthermore, the number of granulocytes in the systemic circulation was significantly increased following bacteremia in wild-type mice (Figure 9C). *Gli1* gene deletion inhibited the increase in granulocytes in the blood stream following systemic *E. coli* infection.

## Discussion

Activation of the SHH–Gli1 signal transduction pathway initiates at the engagement of SHH ligand to its receptors (PTCH 1 and 2) on the surface of cells (31). Our current study revealed that *E. coli* bacteremia significantly upregulated SHH expression by bone marrow hematopoietic cells. Twenty-four hours post intravenous challenge with *E. coli*, SHH protein expression by arrow lin^+^ and lin-c-kit^+^ cells was significantly upregulated. Furthermore, marrow lin^+^, lin^−^, and lin-c-kit^+^ cells all exhibited a significant increase in SHH expression at 48 h of *E. coli* bacteremia. This increase in SHH expression by marrow hematopoietic cells may support the activation of SHH signaling in the SHH responding cells. Since lin^+^ cells constitute the major hematopoietic cell population in the bone marrow. They apparently serve as the predominant resource for producing SHH ligand in the bone marrow. An interesting observation was that in contrast to the significant increase in the level of cell-associated SHH, the soluble SHH level in the bone marrow reduced markedly following *E. coli* bacteremia. Therefore, the potential for soluble SHH ligand to activate the SHH signal pathway in distant cells seems to be restricted in this circumstance. Rather, the direct contact between adjacent cells expressing SHH ligand and the correspondent receptors, respectively, is likely critical for effective activation of SHH signaling in the SHH responding cells following systemic bacterial infection. Studies have shown that primitive hematopoietic precursor cells, such as HSCs and upstream progenitors, preferentially lodge in their specific niche compartment relatively separating from sites for storing more mature cells in the bone marrow (32). Even within the HSC population, those maintaining the status of dormancy are believed to reside in the unique niche structures differing from places housing their counterparts undergoing proliferation in the bone marrow (33). Take these facts into consideration, it is likely that the enhanced expression of SHH by primitive hematopoietic precursor cells may play an important role in initial activation of SHH signaling in themselves. The upregulated expression of SHH by more mature lin^+^ hematopoietic cells may provide continuing support to activation of SHH signaling in lin-committed precursor cells during the granulopoietic response to systemic *E. coli* infection. In this study, changes in SHH expression were analyzed primarily in marrow hematopoietic cells. Bone marrow niche environment contains other cell types, including osteoblast cells, stromal cells, and endothelial cells. Further investigation on the alteration of SHH expression by these niche cell types will be helpful for further elucidating their possible involvement in the regulation of SHH signaling in hematopoietic precursor cells during the host response to systemic bacterial infection.

In this study, we observed that SHH mRNA expression by nucleated BMCs was significantly upregulated during the early stage of *E. coli* bacteremia, indicating the involvement of transcriptional regulation in the enhancement of SHH expression. To delineate the underlying signaling mechanism, we analyzed murine *shh* promoter sequence using the AliBaba2.1 database. Fourteen SP1 transcription factor binding sites with the identity of sequence greater than 80% were detected. Previous studies have shown that hematopoietic cells including HSCs express full profiles of TLRs (34). Ligand (such as LPS) binding to TLR4 activates the Ras-c-Raf-mitogen-activated protein kinase ERK kinase 1/2 (MEK1/2) system which in turn activates the ERK1/2 signaling component (35, 36). Phosphorylation of ERK1/2 promotes its nuclear translocation to activate SP1 expression (29, 30). In the current study, we observed that the level of phospho-ERK1/2 in BMCs was markedly increased at 8 h of bacteremia. SP1 expression in marrow lin^+^, lin^−^c-kit^+^, and LKS cells was significantly increased 18 h following *E. coli* infection. Inhibition of ERK1/2 activation with specific MEK1/2 inhibitor PD98059 abolished LPS-stimulated increase in SHH expression by cultured BMCs. Furthermore, reporter gene analysis demonstrated that SP1 markedly activated murine SHH promoter. These results demonstrate that the TLR4–ERK1/2–SP1 signal pathway plays a key role in the regulation of SHH expression by hematopoietic cells in the bone marrow during the host response to bacteremia.

Gli1 is a key transcription factor in the SHH signal pathway, activation of which promotes expression of downstream gene products for programming cell fate and modulating cell activities (17–23). We determined the level of Gli1 expression by BMC subtypes at different stages of differentiation in control mice. Under homeostatic circumstance, the marrow LKS cells expressed the highest level of Gli1. Gli1 expression reduced with the maturation of hematopoietic cells. The lowest level of Gli1 expression was seen in Lin^+^ cells. These observations suggest that primitive hematopoietic precursor cells, particularly HSCs (LKS cells), have a strong activity of SHH signaling in normal state. Our data support the significant role of SHH signaling in the regulation of HSC and upstream progenitor cell function under homeostatic condition. Previous studies have shown that SHH promotes primitive hematopoietic precursor cell proliferation and their myeloid differentiation (8, 24, 25). With cell differentiation into the mature stage, the SHH signaling activity reduces. This observation suggests that mature hematopoietic cells might no longer heavily rely on SHH signaling for the regulation of their functional activities in the homeostatic state.

In response to systemic *E. coli* infection, marrow hematopoietic cells significantly increased Gli1 expression in our current study. In marrow lin^−^ hematopoietic precursor cell fraction, LKS cells were most active in increasing Gli1 expression. *E. coli* bacteremia caused a marked increase in both the number of Gli1^+^LKS cells and the level of Gli1 protein expression by LKS cells. These data demonstrate that marrow primitive hematopoietic precursor cells possess a substantial capacity to actively respond to enhanced stimulation of SHH ligand in their niche environment during the host response to an infectious challenge. Although the percentage of Gli1^+^ cells in the LKS^−^ subpopulation and the level of Gli1 protein expression by LKS^−^ cells increased, the total number of Gli1^+^ LKS^−^ cells in BMCs significantly reduced by 48 h following bacteremia. This observation suggests that SHH signaling is also activated in LKS^−^ cells following systemic *E. coli* infection. The reduction in the number of Gli1^+^ LKS^−^ cells in BMCs is likely caused by conversion of LKS^−^ cells to cells no longer belonging to the LKS^−^ phenotype. Our previous investigations have shown that re-expression of Sca-1 by LKS^−^ cells leads to phenotypic conversion of LKS^−^ cells to LKS cells, which play an important role in the rapid expansion of LKS cell pool in the bone marrow during the host response to serious bacterial infection (2, 3, 37). It is apparent that the phenotypic conversion of Gli1^+^ LKS^−^ cells may also contribute to the marked increase in the number of Gli1^+^ LKS cells in BMCs following bacteremia in our current study. Transcriptional activation is involved in the increase in Gli1 expression by hematopoietic precursor cells in response to *E. coli* infection. In our current study, Gli1 mRNA expression was significantly upregulated in lin^−^c-kit^+^ cells at 24 h following bacteremia. The enhancement of Gli1 expression was also detected in marrow lin^+^ cells during systemic *E. coli* infection in this study. This increase in Gli1 expression in marrow lin^+^ cells appears to imply that the activation of the SHH signaling may also participate in the regulation of functional activities in lin-committed hematopoietic progenitors during the granulopoietic response to the systemic bacterial infection.

The HH signal pathway is a developmentally conserved regulator for stem cell activities. Studies have shown that HH signaling is involved in the regulation of adult HSC function. Neutralization of HH ligands with specific antibodies inhibits cytokine-induced proliferation of primitive human hematopoietic cells (8). Conversely, SHH treatment induces the expansion of pluripotent human hematopoietic repopulating cells in the recipient immunodeficient mice. Downstream activation of the HH signal pathway induces expansion of primitive bone marrow hematopoietic cells under homeostatic conditions and during acute regenerative response to 5-fluorouracil-induced ablation of actively cycling cells in the hematopoietic system (7). Deletion of *Gli1* in mice causes accumulation of more quiescent HSCs as well as decrease in myeloid development (25). In the current study, we examined the role of SHH–Gli1 signaling in the activation of primitive hematopoietic precursor cells during the granulopoietic response to system bacterial infection. The results showed that the increase in proliferative activity (as reflected by cell BrdU incorporation) in LKS cells following bacteremia was essentially in those cells expressing Gli1. By contrast, Gli1^−^LKS did not exhibit any activation of proliferation. Furthermore, *in vitro* stimulation with recombinant murine SHH ligand significantly increased CFU-GM forming activity of sorted lin^−^c-kit^+^ cells. These data indicate that the SHH–Gli1 signal pathway participates in mediating primitive hematopoietic precursor cell activation and reprograming for enhancing their commitment toward granulocyte lin development.

Employing an *in vivo* murine model with *Gli1* deletion, we further verified the role of SHH–Gli1 signaling in mediating primitive hematopoietic precursor cell activation during the granulopoietic response to systemic *E. coli* infection. Our data showed that bacteremia-induced activation of proliferation in primitive hematopoietic precursor cells was significantly attenuated in mice with *Gli1* deletion. Accompanied with the inhibition of primitive hematopoietic precursor cell activation, the increase in the number of granulocytes in the systemic circulation during the host response to *E. coli* bacteremia was also inhibited in mice with *Gli1* gene deletion. Our results from the current investigation demonstrate that activation of SHH–Gli1 signaling plays a pivotal role in mediating primitive hematopoietic precursor cell activation during the granulopoietic response to serious bacterial infection.

## Ethics Statement

This study was carried out in accordance with the recommendations of National Institutes of Health guidelines. The protocol was approved by the Institutional Animal Care and Use Committees of Northeast Ohio Medical University and Michigan State University prior to initiation of all experiments.

## Author Contributions

XS: substantial contributions to concept, designing and conducting experiments, data acquisition, analysis, and interpretation; participating in drafting the work; final approval of the version for publication; agreement to be accountable for all aspects of the work in ensuring that questions related to the accuracy or integrity of any part of the work are appropriately investigated and resolved. SW, KS, DC, and TE: substantial contributions to conducting experiments, data acquisition and analysis; participating in drafting the work; final approval of the version for publication; agreement to be accountable for all aspects of the work in ensuring that questions related to the accuracy or integrity of any part of the work are appropriately investigated and resolved. PZ: substantial contributions to the conception and design of the work; conducting experiments, data acquisition, analysis, and interpretation; drafting the work; final approval of the version for publication; agreement to be accountable for all aspects of the work in ensuring that questions related to the accuracy or integrity of any part of the work are appropriately investigated and resolved, financially responsible for the entire study.

## Conflict of Interest Statement

The authors declare that the research was conducted in the absence of any commercial or financial relationships that could be construed as a potential conflict of interest.
